# Exploring the Connection Between Domestic Violence and Masticatory Outcomes in the Pediatric Population: A Systematic Review

**DOI:** 10.7759/cureus.46764

**Published:** 2023-10-09

**Authors:** Jyotsna Kaur Girgla, Suganya Mahadeva, Madhulika Srivastava, Lokesh Sharma, Sameer Kedia, Sinam Subhaschandra Singh

**Affiliations:** 1 Dentistry, Shiromani Gurudwara Prabandhak Committee, Sri Amritsar, Amritsar, IND; 2 Department of Pedodontics and Preventive Dentistry, People's College Of Dental Science and Research Center, People's University, Bhopal, IND; 3 Department of Pedodontics and Preventive Dentistry, Manav Rachna Dental College, Faridabad, IND; 4 Public Health Dentistry, Maharana Pratap Dental College, Kanpur, IND; 5 Periodontics, VYWS (Vidarbha Youth Welfare Society) Dental College and Hospital, Amaravati, IND; 6 School of Dentistry, Karnavati University, Gandhinagar, IND; 7 Dental College, Regional Institute of Medical Sciences, Imphal, IND

**Keywords:** bruxism, temporomandibular disorders, oral health, family violence, child abuse, domestic abuse

## Abstract

The potential interplay between domestic violence and masticatory outcomes in children and adolescents has garnered increasing attention. Understanding the association between domestic abuse and specific oral health parameters, such as biting habits, temporomandibular disorders (TMDs), and bruxism, holds implications for holistic healthcare interventions. This systematic review aims to synthesize the available evidence to elucidate the potential relationships between domestic abuse and targeted oral health outcomes in the pediatric population. A comprehensive search strategy was conducted across eight databases, namely, PubMed, Embase, Scopus, PsycINFO, Web of Science, Cumulative Index of Nursing and Allied Health Literature (CINAHL), Cochrane Library, and Google Scholar. Boolean operators and Medical Subject Headings (MeSH) keywords were strategically employed to optimize search precision. Clinical studies investigating the relationships between domestic abuse and TMDs, or bruxism, in children and adolescents were included. Two reviewers extracted the data independently. The methodological quality and risk of bias of the selected studies were critically appraised using the Newcastle-Ottawa scale. The systematic search identified three papers investigating the associations between domestic abuse and the targeted oral health parameters. Children in the age group of 6-19 years were assessed. The synthesized evidence revealed a consistent association between domestic abuse and masticatory outcomes. Individuals subjected to domestic abuse exhibited a greater percentage of masticatory anomalies. The methodological assessment of the included studies showed good quality. This systematic review provides a notable synthesis of evidence regarding the associations between domestic abuse and masticatory outcomes in children and adolescents. The complex nature of these relationships necessitates further research to unravel the underlying mechanisms and establish causality. The insights from this review highlight the significance of integrating abuse assessment within oral health evaluations and underscore the need for interdisciplinary collaborations to address the potential impact of abusive experiences on the oral health and well-being of the pediatric population.

## Introduction and background

Domestic abuse is a public health issue that has serious effects on children's well-being [1]. It includes physical, emotional, sexual, and psychological mistreatments that occur in familial or domestic settings. Literature evidence documents the effects of domestic abuse on children's mental, emotional, and physical health [2]. The relationship between domestic abuse and masticatory anomalies has remained unexplored despite the links between bad childhood experiences and long-term health outcomes being well established [2].

Children's dental health conditions have a significant impact on their nutrition, speech, and self-esteem, as well as their general health and quality of life (QoL). It is crucial to look at any connections between domestic abuse and dental health outcomes because of how susceptible children are to their immediate surroundings [3]. For healthcare professionals, policymakers, and child welfare activists alike, it is crucial to understand whether and how domestic abuse affects oral health indicators, such as dental caries, malocclusion, and oral health-related quality of life (OHRQoL) [4].

Child maltreatment is a widespread issue that demands in-depth investigation. It includes a variety of physical, emotional, sexual, and neglectful acts that have the potential or actual to harm a child's health, developmental trajectory, survival, and dignity within the context of power dynamics or relationships [5]. Given India's demographics as a country with roughly 19% of the world's children, 18% of whom are under the age of 18, the vulnerability of children and adolescents to abuse has come to the forefront of social concern [6]. Because of their psychological reliance, these young people are vulnerable to mistreatment, demanding a comprehensive understanding of the characteristics of child abuse and neglect (CAN) [7].

Across organizational and professional barriers, a collective, communal effort is required to address the complicated problem of child abuse [8]. The crucial role played by healthcare professionals, especially dentists, in spotting abuse and neglect serves as a reminder of this responsibility [9]. Dental professionals are well positioned to play a key role in early detection given that a significant 50-67% of physical injuries appear in the oro-facial domain and are assessable [9-10]. Notably, impaired dental health is a typical sign of child neglect, emphasizing the importance of healthcare professionals in picking up on subtle clues.

Studies that assess the effect of CAN on masticatory anomalies are scarce. In fact, the literature on CAN and its impact on oral health itself are negligible. Improving medical education's ability to handle CAN is essential for raising awareness and competency in spotting and reporting cases of abuse [11]. Unfortunately, international research reveals a worrying pattern in which healthcare professionals fail to disclose suspected cases of abuse for a variety of reasons, including a lack of awareness, sociocultural pressures, and structural flaws [7,10-12]. To protect the welfare of the most vulnerable members of society, the panorama of child maltreatment thus demands a radical shift that crosses professional disciplines. The primary goal of this systematic review was to thoroughly examine and evaluate the body of literature that already existed regarding the relationships between domestic abuse and masticatory outcomes in the pediatric population, such as temporomandibular disorders (TMDs), biting habits, and bruxism.

## Review

Materials and methods

Eligibility Criteria

The present review adhered to the Preferred Reporting Items for Systematic Reviews and Meta-Analyses (PRISMA) framework [13] so as to ensure a systematic and thorough approach. This review was conducted following a well-structured PECO (Population, Exposure, Comparator, and Outcomes) protocol to answer the research question, "What is the relationship between domestic abuse and masticatory outcomes encompassing TMDs and bruxism in children and young adults?"

Population (P): The target population consisted of children and adolescents ranging from 0 to 19 years of age, regardless of gender or geographical location.

Exposure (E): The exposure of interest here is to domestic abuse, encompassing physical, emotional, psychological, or sexual abuse within family or domestic contexts.

Comparator (C): No comparable group was included.

Outcome (O): The primary outcome of interest were masticatory anomalies that included TMDs and bruxism. TMDs encompassed a range of temporomandibular joint and muscle-related disorders arising due to abusive incidents. Bruxism, defined as the involuntary grinding or clenching of teeth, was explored as a potential consequence of domestic abuse.

To ensure the selection of pertinent and appropriate papers for analysis, this systematic review used a predefined set of inclusion and exclusion criteria. Included studies were (1) studies focused on the association between domestic abuse (inclusive of physical, emotional, sexual, or psychological abuse and neglect) and oral health outcomes in children aged 0 to 19 years; (2) peer-reviewed studies employing cross-sectional or case-control designs. Studies were excluded if they did not meet the aforementioned inclusion criteria or if they fell under any of the following categories: (1) studies that primarily focused on adult populations or did not explicitly investigate the specified age range of 0 to 19 years; (2) studies that employed intervention-based methodologies; (3) studies that were published as abstracts, conference proceedings, editorials, or reviews without original data; and (4) studies not accessible in full-text form.

Search Strategy

An extensive search was conducted across eight databases to identify pertinent studies. The search strategy encompassed the utilization of Boolean operators (AND, OR) and MeSH (Medical Subject Headings) keywords to optimize search precision. The databases searched included PubMed, Embase, Scopus, PsycINFO, Web of Science, Cumulative Index of Nursing and Allied Health Literature (CINAHL), Cochrane Library, and Google Scholar. The Boolean operators "AND" and "OR" were strategically employed to combine MeSH terms and keywords, enhancing the search's specificity and inclusivity. The primary search terms included variations of "domestic abuse," "family violence," "child abuse," "oral health," "dental trauma," "temporomandibular disorders," and "bruxism." MeSH terms were incorporated when available, enabling standardized terminology usage. Gray literature was also explored for any literature available. The search strategy aimed to capture literature relevant to the intersection of domestic abuse and oral health outcomes in the defined population (Table 1).

**Table 1:** Search strategy implemented across different databases for this review

Database	Search String
PubMed/MEDLINE	("Domestic Violence"[Mesh] OR "Child Abuse"[Mesh] OR "Family Violence"[Mesh] OR "Intimate Partner Violence"[Mesh]) AND ("Oral Health"[Mesh] OR "Dental Trauma"[Mesh] OR "Temporomandibular Joint Disorders"[Mesh] OR "Bruxism"[Mesh]) AND ("Child"[Mesh] OR "Adolescent"[Mesh] OR "Pediatrics"[Mesh])
Embase	('domestic violence'/exp OR 'child abuse'/exp OR 'family violence'/exp OR 'intimate partner violence'/exp) AND ('oral health'/exp OR 'dental trauma'/exp OR 'temporomandibular joint disorders'/exp OR 'bruxism'/exp) AND ('child'/exp OR 'adolescent'/exp OR 'pediatrics'/exp)
Scopus	(TITLE-ABS-KEY("domestic violence") OR TITLE-ABS-KEY("child abuse") OR TITLE-ABS-KEY("family violence") OR TITLE-ABS-KEY("intimate partner violence")) AND (TITLE-ABS-KEY("oral health") OR TITLE-ABS-KEY("dental trauma") OR TITLE-ABS-KEY("temporomandibular joint disorders") OR TITLE-ABS-KEY("bruxism")) AND (TITLE-ABS-KEY("child") OR TITLE-ABS-KEY("adolescent") OR TITLE-ABS-KEY("pediatrics"))
PsycINFO	("domestic violence" OR "child abuse" OR "family violence" OR "intimate partner violence") AND ("oral health" OR "dental trauma" OR "temporomandibular joint disorders" OR "bruxism") AND ("child" OR "adolescent" OR "pediatrics")
Web of Science	TS=("domestic violence" OR "child abuse" OR "family violence" OR "intimate partner violence") AND TS=("oral health" OR "dental trauma" OR "temporomandibular joint disorders" OR "bruxism") AND TS=("child" OR "adolescent" OR "pediatrics")
CINAHL	("domestic violence" OR "child abuse" OR "family violence" OR "intimate partner violence") AND ("oral health" OR "dental trauma" OR "temporomandibular joint disorders" OR "bruxism") AND ("child" OR "adolescent" OR "pediatrics")
Cochrane Library	("domestic violence" OR "child abuse" OR "family violence" OR "intimate partner violence") AND ("oral health" OR "dental trauma" OR "temporomandibular joint disorders" OR "bruxism") AND ("child" OR "adolescent" OR "pediatrics")
Google Scholar	intitle:"domestic violence" OR intitle:"child abuse" OR intitle:"family violence" OR intitle:"intimate partner violence" AND intitle:"oral health" OR intitle:"dental trauma" OR intitle:"temporomandibular joint disorders" OR intitle:"bruxism" AND intitle:"child" OR intitle:"adolescent" OR intitle:"pediatrics"

Data Extraction

A careful data extraction strategy was framed to ensure the reliable retrieval of pertinent data from the chosen research. The screening of articles and data extraction technique involved two independent reviewers. Data were extracted under the categories of study ID, publication year, study location, sample size, age ranges, gender ratio, masticatory outcome assessed, and inferences. The Cohen's kappa coefficient was used to evaluate the inter-rater reliability, and the results showed that the two reviewers had a high degree of agreement (0.88). Consensus meetings were used to settle differences in the data extraction, and a third reviewer was consulted to clarify any remaining doubts by one-to-one discussions.

Risk-of-Bias Assessment

The Newcastle-Ottawa Scale (NOS) tool [14] was used to assess the quality of the included studies in the review. The NOS assigns stars or points to various aspects of a study, allowing for a systematic and transparent assessment of study quality. The NOS is divided into three main components or domains, each of which assesses a different aspect of study quality. The selection domain evaluated the representativeness of the study population, the ascertainment of exposure or intervention, and the demonstration that the outcome of interest was not present at the start of the study. More stars indicate a better-quality study. The comparability domain assesses the comparability of study groups or cohorts. The outcome domain examined the assessment of the outcome of interest, including the follow-up duration and whether the outcome was assessed in an objective and valid way. The NOS assigns a maximum of nine stars to each study: four stars for selection, two stars for comparability, and three stars for outcome. A higher total score indicates a better methodological quality. For each chosen study, the bias assessment process was carried out independently by the same two reviewers. Consensus meetings were used to settle any disagreements in the evaluation or, if necessary, in consultation with a third reviewer.

Results

Study Characteristics

**Figure 1:**
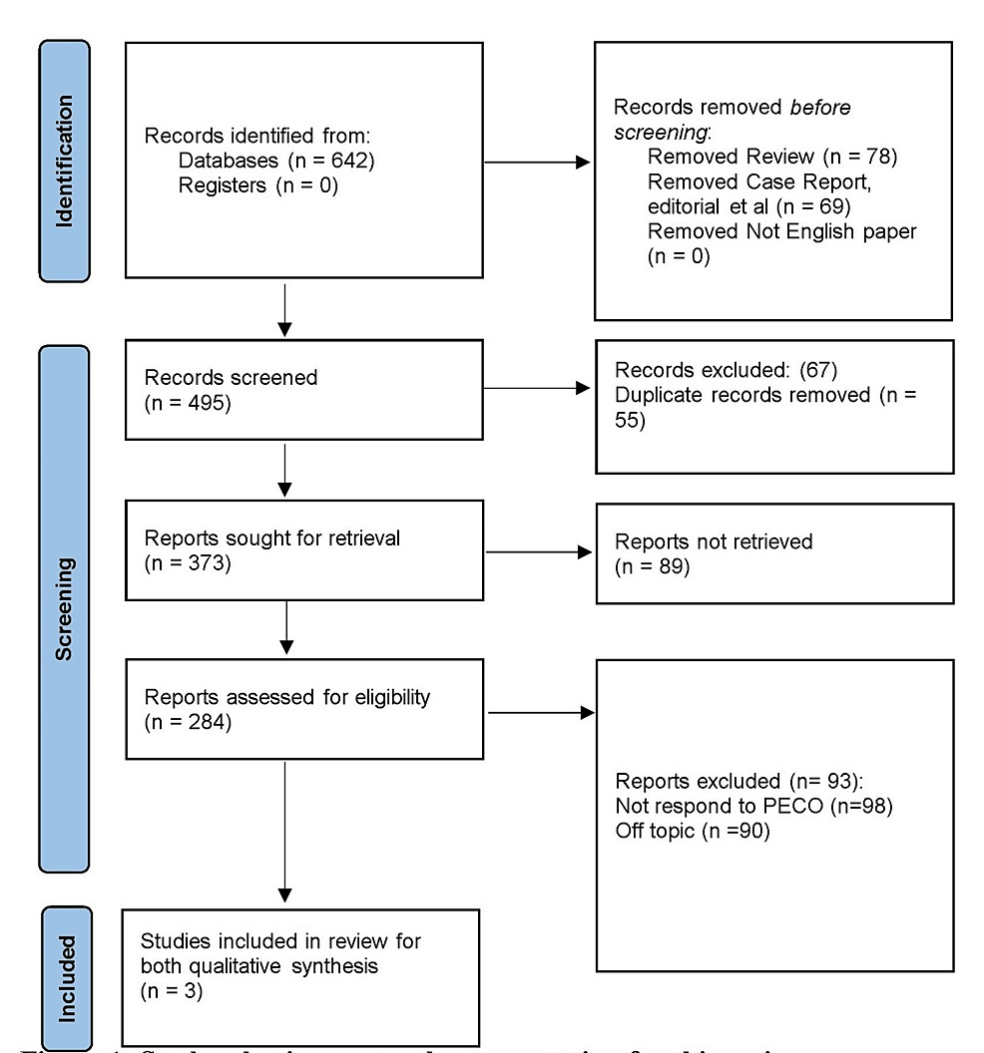
Study selection protocol representation for this review

A total of 642 items were located in the searched databases (PubMed = 302, Embase = 28, Scopus =103, PsycINFO = 26, Web of Science =113, CINAHL = 29, Cochrane Library = 0, and Google Scholar = 41). Gray literature did not yield any records. Among the discovered records, 78 were excluded as reviews, and 69 were further eliminated as case reports or editorials. Fifty-five were duplicate records, which were removed. Finally, three papers [15-17] were selected for the qualitative analysis, as shown in Figure 1.

The selected papers [15-17] shown in Table 2 collectively offer a thorough summary of the relationships between abusive events and masticatory outcomes in children and young adolescents. Two studies [15,16] were conducted in Brazil and one in Mongolia [17]. The sample size ranged from 104 to 2431. The study population ranged in age from 6 to 19 years. Papers eligible to be included were all published in the last decade.

**Table 2:** Studies selected for the review and their associated assessment TMD: temporomandibular disorder

Study/year	Year	Origin	Study protocol	Sample size (n)	Age range assessed (in years)	Masticatory outcome/ parameters assessed	Main outcomes
Martinez et al. [15]	2016	Brazil	Case control	104	6-16	Bruxism and clenching habits	Abused children exhibited bruxism in 19% of cases and clenching in 57% as compared to 7.7% bruxism and 21.2% clenching in non-abused children.
Nascimento et al. [16]	2021	Brazil	Cross-sectional	2431	14-19	TMDs	40% of the respondents reported having a positive response for TMDs as a result of abuse episodes; 31.2% reported pain in the temple region; 8.9% had tenderness during jaw movement.
Okawara et al. [17]	2022	Mongolia	Cross-sectional	770	11.8 + 1.9	Bruxism and biting habits (lip, nail, and pencil)	17.5% of the respondents reported bruxism. The adjusted model for age, gender, and socioeconomic status showed no association for either physical or psychological abuse. Biting habits was found to be significantly associated with abuse.

Main Findings

The synthesis results demonstrated that children subjected to domestic abuse were likely to be associated with higher percentages of masticatory anomalies. Despite the variations in sample sizes, origins, and evaluation criteria among the studies, several overall tendencies may be found. The age range of the individuals also appears to have an impact on the relationships that were found. Examinations of younger children suggest a possible link between oral trauma and abusive events. Studies involving older teenagers, however, place more emphasis on connections with TMDs. This age-related variation in relationships may be explained by changes in how susceptible different age groups are to particular oral health conditions brought on by abusive events.

Risk-of-Bias Assessment

All three studies were of moderate quality, with scores ranging from 5 to 8, as shown in Table 3. Less stars were assigned for the outcome domain in all studies, as none of them evaluated masticatory anomalies in an objective manner.

**Table 3:** Risk-of-bias assessment for the studies included

Study ID	Is the case design adequate?	Selection			Comparability of cases and controls	Outcome			Total
		Case representativeness	Control selection	Definition of controls		Ascertainment of exposure	Same method of ascertainment	Non response	
Martinez et al. [15]	*	*	*	*	*	*	*	*	8
Nascimento et al. [16]	*	*	-	-	-	*	*	*	5
Okawara et al [17]	*	*	-	-	-	*	*	*	5

Discussion

The current review demonstrated the synthesized evidence of an association between domestic violence and masticatory anomalies in pediatrics. Masticatory anomalies noted were bruxism and symptoms of TMDs. Although an association was noted, it is essential to comprehend the underlying mechanisms and pathways linking domestic violence and masticatory anomalies. The literature shows that stress, anxiety, and trauma resulting from domestic violence may lead to parafunctional oral habits, altered muscle activity, and changes in craniofacial development. Domestic violence can have a profound psychological impact on children, including increased stress and anxiety levels. These psychological consequences may manifest in oral habits, such as teeth grinding (bruxism) and clenching, which, over time, can contribute to masticatory anomalies.

The study of Martinez et al. [15] showed that females suffered greatly from abuse as compared to their male counterparts. This could be attributed to their submissive stance in the family environment. The condition could also be aggravated due to psychosocial components and hormonal influences during puberty. The study of Nascimento et al. [16] was a school-based study and included samples from low and middle socioeconomic status. The questionnaire developed to record self-reported symptoms for TMDs was validated. Although the study established an association between abuse and self-reported TMD symptoms, the multifactorial nature of TMDs cannot be overlooked. An element of recall bias should also be considered, as the participants self-reported TMD symptoms. The study by Okawara et al. [17] might have shown underreporting of bruxism as it occurs during the night and hence could not have been recognized. Bruxism was not confirmed either by polysomnography or electromyography highlighting the lack of objective measures. None of the studies reported on the timing and duration of abuse.

The literature shows several studies on domestic abuse in children with other dental outcomes that need to be mentioned. Valencia-Rojas et al. [18] performed a cross-sectional study in Canada, involving a smaller sample of 66 children aged two to six years, to assess dental trauma. A proportion (6%) of children reported an incidence of dental trauma with a history of abuse. Bright et al. [19] conducted a cross-sectional study in the USA, involving a vast sample size of 90,555 individuals aged 0-17. Their findings revealed a graded correlation between the likelihood of carers reporting fair to poor oral health and the extent of abuse faced. Da Silva et al. [20], in a Brazilian cross-sectional investigation with 192 participants aged eight to 10, demonstrated that victims of child abuse exhibited worse levels of oral symptom scores, underscoring the detrimental impact on OHRQoL. Duda et al. [21], in case-control study in Brazil conducted on 240 participants (122 cases), revealed that the case group had a significantly greater mean decayed, missing, and filled tooth (DMFT) score and unmet dental needs in comparison to controls. The studies of Greene et al. [22] and Greene P et al. [23] conducted in the USA demonstrated association between abuse and compromised oral health outcomes, with children abused being more likely to exhibit poorer DMFS or untreated decay. A cross-sectional study done by Gurunathan et al. [24] conducted in India on 478 participants linked certain demographic groups and individuals with infrequent dental care utilization to higher dental neglect scores. Through these varied methodologies and contextual lenses, these studies illuminate correlations between abuse and various oral health indicators, enriching the scientific discourse and emphasising the importance of holistic interventions for children's well-being.

CAN has long-lasting effects on the physical and mental health of children [25]. Orofacial injuries, including burns in the mouth, lips, tongue, cheeks, maxilla, mandible, and maxillary labial frenum, are also reported. Notably, these wounds ring out as telltale signs of CAN, strengthening the position of dentists in terms of identification and treatment [26]. However, a troubling global tendency shows that dentists frequently fail in their crucial role in the identification of CAN [27-28]. Reports in a paper revealed that both medical and dental residents showed decreased awareness regarding the legislative framework associated with child protection laws [29]. However, another report revealed a serious lack of understanding among dental professionals, with 45% showing insufficient understanding of referral protocols for CAN [30]. An unsettling truth is that perpetrators frequently take on the personas of people they know. Making sure healthcare professionals are aware of this aspect becomes essential to preventing possible abuse perpetrators [31]. The fear of familial retaliation emerges as a significant barrier to referral as well. Dentists should pay special attention to injuries that do not match the explanation or the child's age and developmental stage. It is also essential to establish a trusting and supportive relationship with children and their guardians. Open communication can enable carers to disclose concerns or information about abuse or neglect.

Limitations

Certain limitations of the review need to be mentioned. The intrinsic variability of the chosen studies in terms of sample characteristics, geographic origins, and assessment procedures can bring significant heterogeneity to the study. As the studies included were case-control and cross-sectional study designs, the ability to demonstrate causal association between domestic abuse and masticatory outcomes is necessarily constrained by these approaches. Moreover, as the data obtained are largely cross-sectional, it is impossible to distinguish between time sequences and determine if abuse directly caused the discrepancies in oral health that have been reported. A meta-analysis could not be performed due to the lack of quantitative scores and availability of very few articles available on the topic.

## Conclusions

The review shows an association between abusive occurrences and masticatory anomalies, which is in line with the available literature. This highlights the physical effects of abuse on oral health, emphasizing the importance of early detection and management. This connection suggests a complex psychophysiological framework that calls for therapeutic approaches that extend beyond simple oral health considerations. It is important to note that existing literature on this topic is scarce, thus emphasizing the need for further studies to comprehensively address the problem. The potential role of collaboration among dentists, pediatricians, and social workers, facilitates the identification and prompt intervention in these cases. Early identification and intervention for children exposed to domestic violence may help mitigate the development of masticatory anomalies and provide holistic support for their well-being.

## References

[1] Leeb RT, Lewis T, Zolotor AJ (2011). A review of physical and mental health consequences of child abuse and neglect and implications for practice. Am J Lifestyle Med.

[2] Rouland B, Vaithianathan R (2018). Cumulative prevalence of maltreatment among New Zealand children, 1998-2015. Am J Public Health.

[3] Sarkar R, Ozanne-Smith J, Bassed R (2021). Systematic review of the patterns of orofacial injuries in physically abused children and adolescents. Trauma Violence Abuse.

[4] Lalor K, McElvaney R (2010). Child sexual abuse, links to later sexual exploitation/high-risk sexual behavior, and prevention/treatment programs. Trauma Violence Abuse.

[5] Tilvawala D, Murray C, Farah R, Broadbent JM (2014). New Zealand dental therapists' beliefs regarding child maltreatment. Aust N Z J Public Health.

[6] Cairns AM, Mok JY, Welbury RR (2005). The dental practitioner and child protection in Scotland. Br Dent J.

[7] Al-Dabaan R, Newton JT, Asimakopoulou K (2014). Knowledge, attitudes, and experience of dentists living in Saudi Arabia toward child abuse and neglect. Saudi Dent J.

[8] Azevedo MS, Goettems ML, Brito A, Possebon AP, Domingues J, Demarco FF, Torriani DD (2012). Child maltreatment: a survey of dentists in southern Brazil. Braz Oral Res.

[9] Herrenkohl TI, Leeb RT, Higgins D (2016). The public health model of child maltreatment prevention. Trauma Violence Abuse.

[10] Uldum B, Christensen HN, Welbury R, Poulsen S (2010). Danish dentists' and dental hygienists' knowledge of and experience with suspicion of child abuse or neglect. Int J Paediatr Dent.

[11] Pietrantonio AM, Wright E, Gibson KN, Alldred T, Jacobson D, Niec A (2013). Mandatory reporting of child abuse and neglect: crafting a positive process for health professionals and caregivers. Child Abuse Negl.

[12] Erisman JC, de Sabbata K, Zuiderent-Jerak T, Syurina EV (2020). Navigating complexity of child abuse through intuition and evidence-based guidelines: a mix-methods study among child and youth healthcare practitioners. BMC Fam Pract.

[13] Page MJ, McKenzie JE, Bossuyt PM (2021). The PRISMA 2020 statement: an updated guideline for reporting systematic reviews. BMJ.

[14] Gierisch JM, Beadles C, Shapiro A (2014). Appendix B: Newcastle-Ottawa scale coding manual for cohort studies. Health disparities in quality indicators of healthcare among adults with mental illness.

[15] Doria Martínez A, Navarro Chong M, Garzón Panesso S (2016). Dental clenching as a sign of child abuse in 6-to-16-year-old institutionalized children [Article in Spanish]. Universitas Odontologica.

[16] Nascimento M, Dahllöf G, Cunha Soares F, Mayer TM, Kvist T, Colares V (2021). Self-reported symptoms of temporomandibular pain and jaw dysfunction in adolescents are associated with exposure to violence. J Oral Rehabil.

[17] Okawara A, Matsuyama Y, Yoshizawa Araki M (2022). Association between child abuse and poor oral habits in Mongolian adolescents. Int J Environ Res Public Health.

[18] Valencia-Rojas N, Lawrence HP, Goodman D (2008). Prevalence of early childhood caries in a population of children with history of maltreatment. J Public Health Dent.

[19] Bright MA, Alford SM, Hinojosa MS, Knapp C, Fernandez-Baca DE (2015). Adverse childhood experiences and dental health in children and adolescents. Community Dent Oral Epidemiol.

[20] da Silva-Júnior IF, Hartwig AD, Stüermer VM, Demarco GT, Goettems ML, Azevedo MS (2018). Oral health-related quality of life in Brazilian child abuse victims: a comparative study. Child Abuse Negl.

[21] Duda JG, Biss SP, Bertoli FM, Bruzamolin CD, Pizzatto E, Souza JF, Losso EM (2017). Oral health status in victims of child abuse: a case-control study. Int J Paediatr Dent.

[22] Greene PE, Chisick MC, Aaron GR (1994). A comparison of oral health status and need for dental care between abused/neglected children and nonabused/non-neglected children. Pediatr Dent.

[23] Greene P, Chisick MC (1995). Child abuse/neglect and the oral health of children's primary dentition. Mil Med.

[24] Gurunathan D, Shanmugaavel AK (2016). Dental neglect among children in Chennai. J Indian Soc Pedod Prev Dent.

[25] Hazar Bodrumlu E, Avşar A, Arslan S (2018). Assessment of knowledge and attitudes of dental students in regard to child abuse in Turkey. Eur J Dent Educ.

[26] Kural D, Abbasoglu Z, Tanboga İ (2020). Awareness and experience regarding child abuse and neglect among dentists in Turkey. J Clin Pediatr Dent.

[27] Nilchian F, Tarrahi MJ, Zare N (2021). A systematic review and meta-analysis of failure to take history as a barrier of reporting child abuse by dentists in private and state clinics. Dent Res J (Isfahan).

[28] Soumya Mohanan TV, Sankeshwari RM, Ankola AV (2020). Perspectives towards child abuse and neglect among dental practitioners in Belagavi city: a cross-sectional study. J Educ Health Promot.

[29] Deshpande A, Macwan C, Poonacha KS, Bargale S, Dhillon S, Porwal P (2015). Knowledge and attitude in regards to physical child abuse amongst medical and dental residents of central Gujarat: a cross-sectional survey. J Indian Soc Pedod Prev Dent.

[30] McGee H, O'Higgins M, Garavan R, Conroy R (2011). Rape and child sexual abuse: what beliefs persist about motives, perpetrators, and survivors?. J Interpers Violence.

[31] Allareddy V, Rampa S, Allareddy V (2017). Opioid abuse in children: an emerging public health crisis in the United States!. Pediatr Res.

